# A Functionalized Membrane Layer as Part of a Dressing to Aid Wound Healing

**DOI:** 10.3390/membranes12100936

**Published:** 2022-09-27

**Authors:** Tomasz Miłek, Anna Grzeczkowicz, Agata Lipko, Leszek Oleksinski, Angelika Kwiatkowska, Marcin Strawski, Monika Drabik, Radosław Stachowiak, Jerzy Goliszewski, Ludomira H. Granicka

**Affiliations:** 1 St. Anna Hospital of Trauma Surgery, Medical Centre of Postgraduate Education, Barska 16/20 St., 02-315 Warsaw, Poland; 2 Nalecz Institute of Biocybernetics and Biomedical Engineering, Polish Academy of Sciences, Trojdena 4 St., 02-109 Warsaw, Poland; 3 Laboratory of Electrochemistry, Faculty of Chemistry, University of Warsaw, 00-927 Warsaw, Poland; 4 Department of Physiology of Bacteria, Faculty of Biology, University of Warsaw, Miecznikowa 1 St., 02-096 Warsaw, Poland

**Keywords:** polyelectrolyte membrane layer, dressing, oxygenating factor, patch, human fibroblasts

## Abstract

Purpose: This study is an approach to a dressing platform based on support functionalized with oxygenating factors within an alginate layer, constituting a safe and even contact surface for interface with a wound. Methods: An alginate layer with incorporated oxygenating elements deposited on the support patch was assessed. As an oxygenating factor, perfluorooctyl was applied, and the layer coatings in two options, cross-linked and not, were evaluated. The function of human dermal fibroblast cells cultured in the presence of these constructs was analyzed, as well as their morphology using flow cytometry, fluorescence microscopy, and scanning electron microscopy. In addition, the membrane coating material was assessed using FTIR, AFM, and SEM-EDX characterization. Results: The applied membrane coatings adsorbed on the patch ensured the viability of the human fibroblasts cultured on the membranes during 10 days of culture. However, on the sixth day of culture, the percentage of live cells grown in the presence of cross-linked alginate with oxygenating factor ((ALG-PFC)net) was significantly higher than that of the cells cultured in the presence of the alginate coatings alone. SEM-EDX analysis of the (ALG-PFC)_net_ confirmed the presence of oxygenating and cross-linking factors. In addition, the regular granular branched structure of the layer coating material involving the oxygenating and cross-linking factors was observed using the AFM technique. Conclusion: The topography of the layer coating material involving the oxygenating and cross-linking factors ensures an even contact surface for interface with the wound. Considering 5-day intervals between dressing replacements, the platform with an oxygenating configuration ensuring the growth and morphology of the human fibroblasts can be recommended at this time as an element of a dressing system.

## 1. Introduction

Wound treatment is a significant problem in modern societies. It is estimated that only in Europe, around 1.5–2 million people suffer from acute and chronic wounds [1]. The situation is aggravated by the fact that the success of applied therapy largely depends on the overall patient’s condition. For example, coexisting diseases worsen wound healing, affecting the effectiveness of treatment. Obesity, diabetes, and other illnesses of civilization have a negative impact, especially on the ageing population of developed countries, where these comorbidities are common. As a result, the clinical and economic burden of treating wounds is increasing worldwide. Although contemporary medicine devotes much attention to minimalizing the risk of complications, the issue of hard-to-heal wounds is still a compelling challenge [2,3]. Furthermore, prolonged treatment resulting from unsuccessful wound healing deteriorates the quality of patients’ lives and, simultaneously, increases the budget of state healthcare institutions [1].

An important aspect of wound healing therapy is optimal treatment with comprehensive dressings and therapies supporting this process [1]. Therefore, scientists conduct intensive research into new materials with specific parameters that can potentially be used in wound dressings. 

A three-dimensional (3D) polymeric network, termed a hydrogel, is commonly applied as a material for dressing. In addition, plant-derived polysaccharide gels such as alginate, chitosan, hyaluronic acid, or gelatin are widely utilized to design dressings for the treatment of non-healing chronic wounds [4,5].

Much attention is given to new polymer composites, encompassing carbon-based nanomaterials, including graphene, graphene oxide, reduced graphene oxide, and graphene quantum dots carbon nanotube [6]. For example, it was shown in vitro that hydrogels based on bacterial cellulose modified with graphene quantum dots could possess antibacterial properties and improve the migration of human gingival fibroblasts [7]. Another example of carbon-based nanomaterial application is a carbon nanotube sponge with hydroxybutyl chitosan for hemorrhage hemostasis, which also showed antibacterial activity [8]. 

Polyelectrolyte membrane coatings are widely used experimentally to improve the surface of biomedical materials. Due to the appropriate components, such systems can be functionalized for selected purposes. For example, polyelectrolyte coatings in cell scaffold systems can be used in cooperation with various cells for dressing applications [9,10]. For example, layered scaffolds made of alginate and poly(L-lysine) enable the functioning of rat neurons during 2-week culture, which may be used in controlled neuronal regeneration [11]. Similarly, the coatings consisting of an alginate and polyethyleneimine layer showed the possibility of maintaining the functions of dental pulp stem cells encapsulated in these coatings [12]. Another important aspect of treating injuries is the involvement of bacteria, which becomes less clear-cut with increasing knowledge of the wound healing process. In order to improve the effectiveness of wound healing and consider the growing multidrug resistance of bacteria, it has become necessary to search for novel, practical materials that can find, among others, applications in systems serving as a dressing, including antibacterial media supporting cell growth. Metal nanoparticles play a specific role here. Since metal nanoparticles can reduce the adhesion of bacterial cells, gold and silver nanoparticles with unique antimicrobial properties in the membrane structure may be considered for use in wound healing dressings. In addition, works have been carried out on developing nanocomposite multifunctional polyelectrolyte layers to obtain a dressing that simultaneously acts as an antibacterial and/or antiviral platform supporting the growth of eukaryotic cells [13]. 

Modern dressing systems are usually based on hydrogels, which ensure biocompatibility and maintain a moist environment, while among ready-made dressings available on the market, mainly two hydrogels are used: alginate and collagen [14,15,16,17]. Membrane functionalization with factors that support wound healing by maintaining cell functioning is an approach that ensures the high efficiency of dressing. As oxygenating factors, perfluorocarbons are highly fluorinated liquids that can act as oxygen carriers for organ preservation in transplantation. Additionally, PFCs can act as ^19^F magnetic resonance imaging agents and oxygen carriers. Thus, constructing the fluorinated carriers will improve their carriers’ stability and endow them with additional functions. Moreover, perfluorocarbon emulsions have been applied to enhance oxygen transport in cell cultures [18]. The PFC influence on cells such as cell line RINm5F beta cells, rat pancreatic cells, A549 cells, and osteoblasts were assessed [19,20,21]. In addition, some authors proposed that the substrates improve oxygenation for the ‘cells’ growth increase. e.g., protein-covered PFC substrates [22]. Photodynamic cancer therapy based on perfluorocarbon nanoemulsions containing fluorinated photosensitizer is another potential application of PFC, which can also act as a ^19^F magnetic resonance imaging agent. PFC-based systems for diagnosis and treatment have been constantly developed [23,24]. Furthermore, perfluorinated compounds, widely used in consumer and industrial products and processes, belong to a class of compounds that tend to persist in the environment. Perfluorinated compounds can be detected, e.g., in different foodstuffs, water, plants, mammals, and humans. Therefore, the toxic effects and the contribution of individual pathways of PFC contamination of humans (such as diet, food contact materials, non-food personal items, and indoor and outdoor air) to total contamination have been investigated [25,26]. Nevertheless, because of the strength of the carbon/fluorine bond, the PFC molecules considering their chemical stability and resistance to biological degradation find biomedical applications. 

Considering the global increase in wound management’s clinical and economic burden, new generations of products functionalized to obtain the properties that can affect the wound-healing process have been developed. Our goal was to build a platform that would meet this demand, built of commercially available dressing support functionalized with highly disposable substrates. 

The study aims to approach a dressing platform based on support functionalized with oxygenating factors. The function of cells cultured in the presence of these constructs was analyzed, as well as their morphology using flow cytometry, fluorescence microscopy, atomic force microscopy, and scanning electron microscopy. 

The alginate, which has a natural ability to create stable gels in reaction with solutions containing divalent cations, was applied as a base material. Its gelling is the effect of a conglomeration of the segments of polyguluronic acid in clusters where calcium ions are scattered in the space between the chains (‘egg-box model’) [27]. 

The platform with an oxygenating factor within cross-linked layer coating in a configuration with a commercially available patch as support has not been reported before for wound dressing applications. 

## 2. Materials and Methods

*Reagents*: 1-Bromoheptadecafluorooctane (Sigma-Aldrich, Munchen, Germany); propidium iodide (Sigma-Aldrich), trypsin EDTA solution C (0.5%), EDTA 0.2% (10X) (Biological Industries, Kibbutz Beit HaEmek, Israel), phosphate-buffered saline (PBS) (Biomed Lublin, Lublin, Poland), MilliQ water; AQUACEL foam, nonadhesive (ConvaTec, UE) *Media*: Fibroblast Growth Medium (FGM) (Sigma-Aldrich, München, Germany).

*Cells*: Human Dermal Fibroblasts (HDF) (Sigma-Aldrich)

*Bacterial cells*: Escherichia coli ATCC 25922

### 2.1. Preparation of Polyelectrolyte Membranes Deposited on the Support AQUACEL Foam

The following solutions were used to modify the patch: 2.2% sodium alginate in PBS; 2.5% calcium chloride in physiological saline; mixture of 1-Bromoheptadecafluorooctane (liquid concentration from the brand bottle) and alginate (2.2%) in a 1:9 volume ratio (ALG-PFC).

### 2.2. Preparation of Coatings

A circular patch with an area of around 1.5 cm^2^ (cut from the brand patch) was covered with the following coatings:

- alginate (ALG)—made of 2.2% sodium alginate;
- alginate cross-linked (ALG)net—made of ALG cross-linked with 2.5% CaCl_2_;
- alginate–perfluorooctyl (ALG-PFC)—made from the mixture ALG-PFC;
- alginate–perfluorooctyl cross-linked ((ALG-PFC) net)—made by cross-linking ALG-PFC using 2.5% CaCl_2_.

### 2.3. Cell Culture

Human Dermal Fibroblast (HDF) cells were maintained under standard culture conditions (5% CO_2_, 37 °C) in Fibroblast Growth Medium. Cells were grown to approximately 80% confluency. The medium was then removed from the culture bottles, whereas the cells were washed with PBS without calcium and magnesium ions and trypsinized. Next, 1 mL of a suspension of HDF cells at a concentration of 5 × 10^3^ was added to the culture wells. After one day, the patch was placed in the well with the modified surface facing down to obtain direct contact with fibroblasts. After 3-, 6-, and 10 days of culture, the supports were removed, the cells in the culture wells were trypsinized, and then, the viability of the cells was examined using propidium iodide in a flow cytometer. As a control, the cells were grown in the presence of unmodified supports.

Additionally, the surface of the glass slices was modified with the coatings. Then, prepared slices were placed in the wells of the culture plate. Next, the HDF cell suspension was added. Cells were grown for 10 days. After 3-, 6-, and 10-day cultivation, the cells were examined using fluorescence microscopy. 

### 2.4. Flow Cytometry 

Flow cytometry was applied to examine the cells’ functioning, wherein data acquisition was performed on the Canto II flow cytometer (Becton Dickinson Immunocytochemistry Systems, Franklin Lake, NY, USA). Then, the results were processed by the FACS Diva software system (Becton Dickinson, Franklin Lake, NY, USA).

### 2.5. MTT Assay 

The (3-4,5-dimethylthiazol-2-yl)-2,5-diphenyltetrazolium bromide (MTT) assay to assess cellular mitochondrial activity was performed. Shortly, the cells were seeded on the membrane films. After 3-, 6-, and 10 days of culture, the MTT solution at the concentration of 5 g/L was added to the culture in a 1:10 dilution of the medium. Next, the cells were incubated for 2 h at 37 °C with 5% CO_2_. Afterward, the solution was discarded, and DMSO was added. After 15 min of shaking, absorbance was measured at 550 nm using a spectrophotometer (HP 8452 diode-array spectrophotometer).

### 2.6. Reactive Oxygen Species (ROS) Assessment in HDF Cells 

The ROS level was determined using a DCFM-DA assay (Invitrogen). After 3-, 6-, and 10 days of culture, the cells were washed with PBS and, at a final concentration of 10^5^ cells/mL, incubated with DCFM-DA for 30 min at 37 °C, protected from light. As a positive control, the cells labeled with DCFMDA were incubated with 50 µM tertbutyl hydrogen peroxide (TBHP) for 4 h. TBHP mimics ROS activity to oxidize DCFDA to fluorescent DCF. Then, the ROS accumulation in cells was assessed using flow cytometry.

### 2.7. Bacterial Strain and Growth Conditions

Bacterial cells were cultivated in LB broth (Biomaxima, Lublin, Poland) at 37 °C with shaking at 120 rpm. Overnight cultures were diluted in fresh medium to an optical density (OD) of 0.05 and transferred to a 24-well plate (0.5 mL per well). 

### 2.8. The Material Influence on Bacterial Proliferation Examination

The material influence on selected bacterial strain proliferation was assessed. The *E. coli* bacterial strain was cultivated in the presence of the evaluated membranes for 24 h. The proliferation of bacterial cells was assessed spectrophotometrically by OD measurement at 600 nm after 24 h of cultivation. During these analyses, only bacteria present in the supernatant were evaluated.

### 2.9. AFM Evaluation

A Multimode 8 Nanoscope atomic force microscope (AFM, Bruker, Billerica, MA, U.S.A.) was used to image the samples’ surfaces. Silicon cantilevers with a spring constant of ca. 5 Nm^−1^ (TapDLC-150, BudgetSensors, Sofia, Bulgaria) were applied for imaging in PeakForce TappingTM microscopy mode. The sample preparation procedure includes the application of 150 µL of solution on the clean mica plate, V1 grade (NanoAndMore GmbH, Wetzlar, Germany). After 10 min, the plates were rinsed with deionized water and dried under a gentle stream of argon. 

### 2.10. Fourier Transform Infrared (FT-IR) Spectroscopy

The infrared spectra were recorded using the Nicolet iS50 FT-IR spectrometer (Thermo Scientific, Waltham, MA, USA) with a DTGS detector. An iTR attenuated total reflection accessory with diamond crystal was used for all experiments. All experiments were performed with a resolution of 4 cm^−1^, and typically, 32 scans were taken for each sample. A 10 µL drop of solution was deposited in the crystal and left to dry under a gentle air stream.

### 2.11. SEM-EDX Evaluation

SEM-EDX characterization was carried out with a Crossbeam 540X (Carl Zeiss Microscopy GmbH, Jena, Germany) scanning electron microscope with an X-FEG cathode. In addition, the EDX maps were collected with the X-MAXN spectrometer (Oxford Instrument, Oxford, UK) operating at 15 keV. Samples were lyophilized before introducing to introduction to the microscope chamber.

### 2.12. Fluorescence Staining

For fluorescence staining, cells immobilized on the layers deposited on glass coverslips were fixed in 4% paraformaldehyde (PFA) in PBS at room temperature (20 °C) for 15 min. Then, cell membranes were permeabilized using TRITON X100 detergent to allow dyes to penetrate individual cells. Then, the fluorochrome-conjugated phalloidin staining F-actin was added. Phalloidin is a toxin isolated from the phylum Amanita fungus (Amanita phalloides), which binds directly to filamentous actin (F-actin) in fibroblasts. After that, the DAPI solution— fluorochrome, specifically staining DNA—was added to the cells to visualize single cells. Under UV light, cell nuclei stained DAPI shows blue fluorescence. After that, the sample was washed in PBS and analyzed in an IX70 fluorescence microscope (Olympus, Tokyo, Japan). The cytoskeleton’s blue DAPI fluorescence (λ = 460–500 nm) of cell nuclei and red phalloidin fluorescence (λ = 570 nm) were assessed. 

### 2.13. Statistical Analysis

The mean values and standard deviations and the significance of difference were calculated using Statistica 7.1 software. Values where *p* < 0.05 were assumed to be significant.

## 3. Results and Discussion 

1-Bromoheptadecafluorooctane, PFC (also called perfluorooctyl bromide or heptadecafluorooctyl bromide) is one of the most broadly analyzed perfluorocarbons. According to Lumb, this 60% emulsion will bring about 50 mL of oxygen per 100 mL on equilibration with 100% oxygen (conditions: atmospheric pressure) [28]. Moreover, it is assumed that perfluorooctyl bromide can dissolve four times more oxygen than CO_2_ [29]. Therefore, in our study, PFC was applied as an oxygenating agent to potentially improve the properties of the designed dressings. ALG-PFC coatings were prepared using the layer-by-layer method. The experiments were also performed for cross*-*linked polyelectrolyte layers—(ALG-PFC)_net_.

### 3.1. Evaluation of the Functioning of Human Fibroblast Cells in the Presence of a Patch Modified with the Membrane Coating Layer 

The developed membrane coatings adsorbed on the support patch were examined for cytotoxicity on the HDF human cell line in vitro. Cells were cultured on modified patches for 10 days. The cells cultured in the presence of patches without membrane coating served as a negative control during 10 days of culture. Cell function and morphology were evaluated quantitatively by flow cytometry and MTT and qualitatively using fluorescent microscopy.

**Figure 1:**
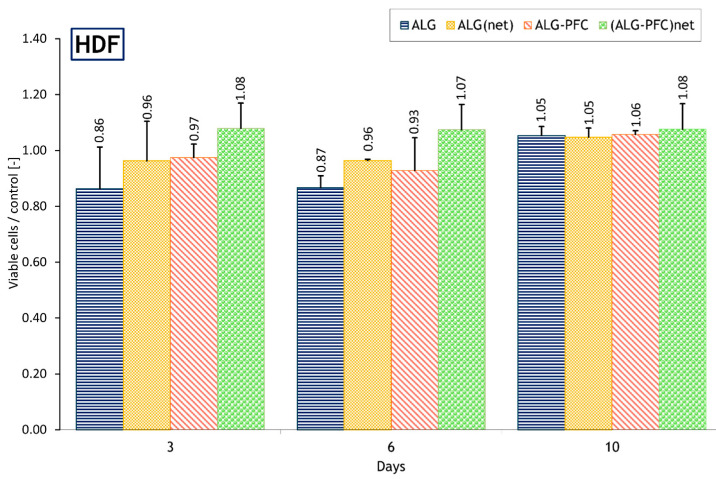
The ratio relative to the control of the percentage of viable HDF cells over 10 days of culture in the absence of patches (control) or the presence of modified patches. Key to the symbols: ALG—alginate, ALG_net_—alginate cross-linked, ALG-PFC—alginate–perfluorooctyl, (ALG-PFC)_net_—alginate–perfluorooctyl cross-linked. The values are presented as mean ± SD (*n* = 6).

To quantitatively assess the condition of cells cultured in the presence of the designed membranes, flow cytometry analysis was performed on the human dermal fibroblasts line (HDF) as a model. The obtained results showed that after 3 days of culture, no significant differences in viability were found between the cells grown in the presence of the patches with tested layers and the control. Furthermore, after 6 days of culture, no significant differences were found between the viability of cells cultured in the presence of ALG, ALG_net_, and ALG-PFC coatings; however, the values of ALG, ALG_net_, and ALG-PFC were significantly lower compared with control. In addition, the percentage of live cells grown in the presence of the (ALG-PFC)_net_ was significantly higher than that of the cells cultured in the presence of the ALG and ALG_net_ coatings. In addition, the ALG and ALG_net_ mean percentage value was higher than the control. Moreover, after 10 days of culture, the values obtained for the cells grown in the presence of all coatings tested were higher compared with control. Finally, no significant differences were found between the viability of cells cultured in the presence of ALG, ALG_net_, ALG-PFC, and (ALG-PFC)_net_ coatings. Therefore, considering 5-day intervals between dressing replacement, the cross-linked coating shell (ALG-PFC)_net_ has been selected for further research. During the modification process with PFC, the hydrolyzable groups react with the hydroxyl groups present in alginate, forming a chemically bonded hydrophobic layer that may impact oxygen transport to the cells. The results of the cytometric test are shown in Figure 1.

To verify the mitochondrial activity of the cells cultured in the presence of the designed surfaces, we applied an MTT assay. 

**Figure 2:**
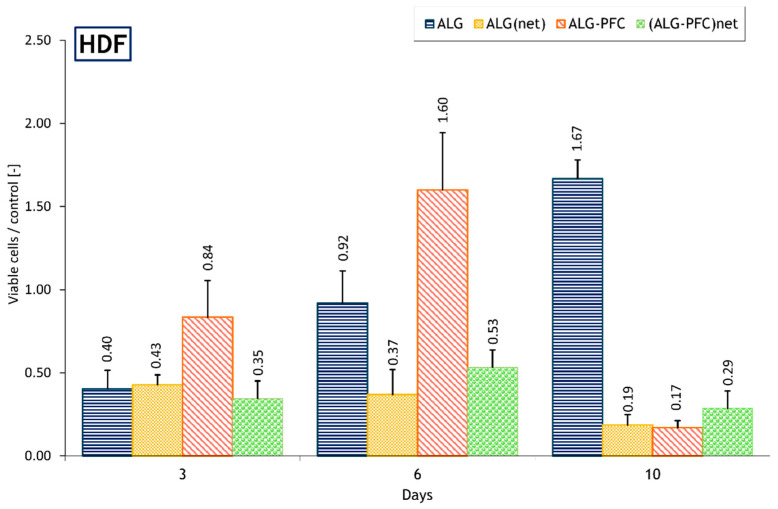
The ratio relative to the control of mitochondrial activity of HDF immobilized on the support with membrane coating represented by formazan production expressed by absorbance during 10-day culture. Key to the symbols: ALG—alginate, ALG_net_—alginate cross-linked, ALG-PFC—alginate–perfluorooctyl, (ALG-PFC)_net_—alginate–perfluorooctyl cross-linked. The values are presented as mean ± SD (*n* = 6).

The presence of PFC within the ALG membrane (ALG-PFC) supports the function of HDF cells after 3 days of culture, achieving values comparable with the control. After 3-day culture on membrane ALG, ALGnet, (ALG-PFC)_net_ meanly 40% of control value was observed, whereas, after 6 days of culture, the ALG membrane maintains HDF ’cells’ function comparable with the control. Moreover, the membrane ALG-PFC exhibit significantly higher mitochondrial activity than the control. The (ALG-PFC)_net_ value was comparable with the value obtained on the third day of culture. On the contrary, the (ALG-PFC)_net_ value was significantly lower than the 6-day value after 10 days of culture (Figure 2). It is true to say that the application of the oxygenating and/or cross-linking factors did not negatively affect HDF cells concerning the viability; however, the fluctuation of mitochondrial activity during the culture can be observed for the support patches with coating involving oxygenating factor and/or cross-linking factor. It can be noted that ROS can affect the MTT results. Mitochondrial metabolism includes the buildup of potentially damaging levels of reactive oxygen species (ROS), Ca^2+^, etc., which must be normalized [30]. Response to stress caused by the introduction of oxygenating and/or cross-linking factors may cause both intra- and inter-mitochondrial redox-environment changes leading to ROS release. The fluctuations of values noticed in the MTT test may, to some extent, reflect it. 

**Figure 3:**
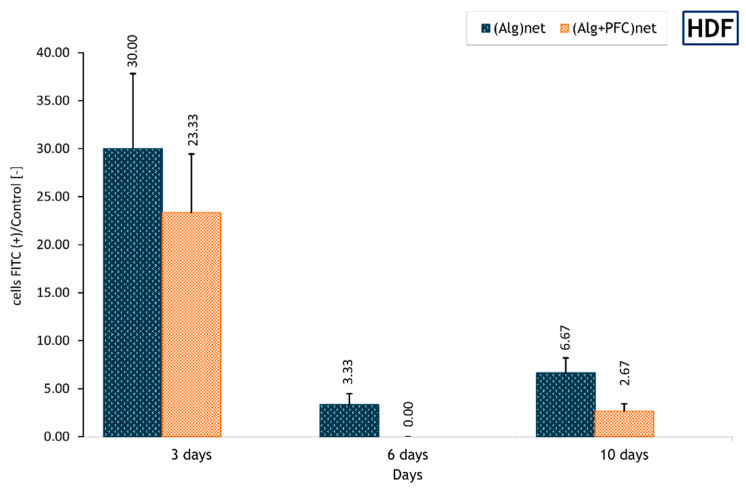
The ratio relative to the control of FITC–positive HDF cells during 10 days of culture. The values are presented as mean ± SD (*n* = 6).

Evaluating the ROS accumulation in cells cultured in cross-linked layer coatings presence using flow cytometry, it was observed that after 3 days of HDF culture in the presence of (ALG)_net_ and (ALG-PFC)_net,_ the share of cells FITC positive was meanly 30% and 23%, respectively, compared with positive control. After 6 and 10 days, this value diminished for (ALG)_net_ to about 3% and 0%, respectively, and for (ALG-PFC)_net_ to 7% and 3%, respectively. The lowest value for (ALG-PFC)_net_ on the sixth day seems to be consistent with the higher value of mitochondrial activity obtained for this coating at that time. (Figure 3).

### 3.2. Evaluation of the Functioning of Microorganisms Cultured in the Presence of the Membranes

**Figure 4:**
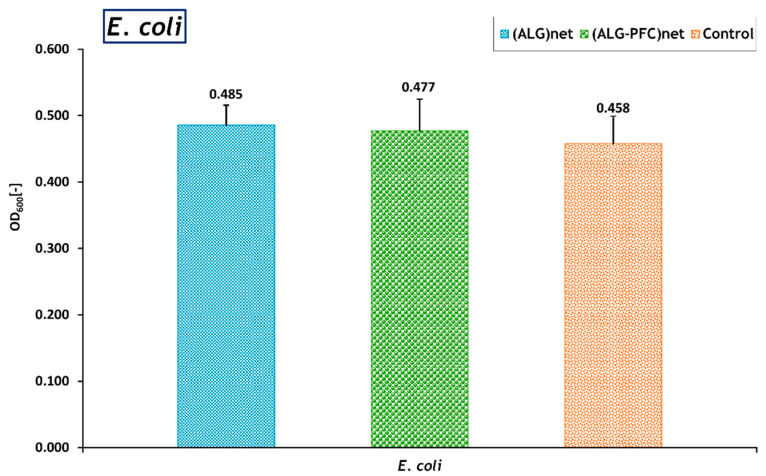
The ratio relative to the control of the optical density (OD) at 600 nm of *E. coli* bacterial strain cultured in the presence of the (ALG-PFC)_net_, (ALG)_net_ (control 2). Key to symbols: (ALG-PFC)_net_—alginate–perfluorooctyl cross-linked; (ALG)_net_—alginate cross-linked; Control—the bacterial strain cultured without the membrane. The values are presented as the mean ± SD (*n* = 6).

The influence of the material on *E. coli* was assessed to analyze whether the (ALG-PFC)_net_ induces the growth of the bacterial cells. Wherein the functioning of bacterial cells cultured for 24 h in the presence of selected membrane ((ALG-PFC)_net_ and ((ALG)_net_) for comparison were investigated. The membrane layer material was added to the bacterial cells suspension in a volume ratio of 1:5. The negative control was bacterial cells maintained in the absence of the membranes. The optical density of the bacterial cell suspension at 600 nm (OD600) was employed to evaluate the function of bacterial cells by applying a TECAN reader, measuring 9 wells. The experiments were carried out in 6 replications. The results of the bacterial function test are presented below (Figure 4).

The evaluated membrane showed no bacteriostatic effect on the tested strain and no induction of bacterial cell proliferation compared to the control (bacterial cells cultured in the absence of the membranes). Furthermore, there were no significant differences in bacteria functioning among cultures in the presence of individual membranes and the absence of membranes (Control). However, some authors [31] observed the antimicrobial influence of alginate oligosaccharides. 

### 3.3. Assessment of the Transport Properties of the Membranes

Some authors have examined diffusion coefficients for the binary aqueous systems containing sodium alginate [32,33,34]. Nevertheless, the systems involving sodium alginate with perfluorooctyl for the same concentrations were not reported. The sodium alginate in PBS dilute solutions, at 2.2%, alone and combined with perfluorooctyl, were evaluated. 

Diffusive permeability was evaluated using a thermodynamic description of diffusive mass transport across a homogenous membrane (Fick’s law) and a two-compartment model [35]; for that purpose, the beads produced from (ALG-PFC)net or (ALG)_net_ (as a control) were placed into the solution of model particles. Dextrans of molecular weight 70 and 150 kDa were applied as the model particles. Then, the concentration of the marker was spectrophotometrically evaluated. 

The control (ALG)_net_ and (ALG-PFC)_net_ adsorbed Dextran 70 (Dex 70), allowing for about 10% release related to the initial concentration value of 1 mg/mL for 30 min. 

The control (ALG)_net_ and (ALG-PFC)_net_ adsorbed Dextran 150 (Dex 150), allowing for about 12% release related to the initial concentration value of 0.250 mg/mL for 30 min, indicating that the evaluated material is permeable for applied markers.

**Figure 5:**
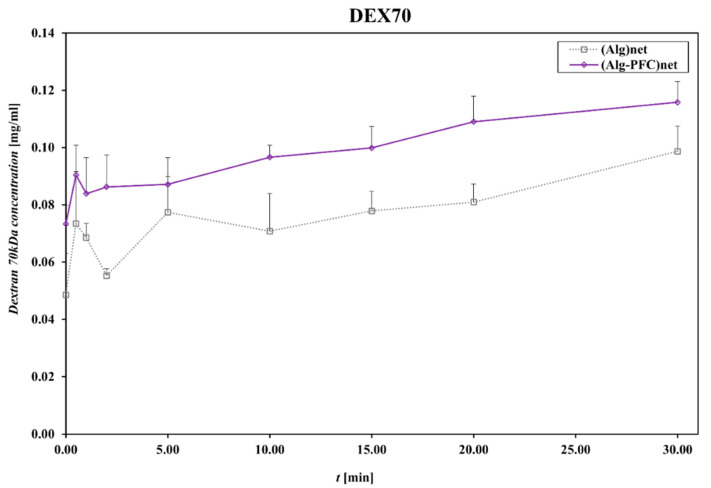
Dextran70 concentration in the function of time during permeation through the membrane. Key to symbols: (ALG-PFC)_net_—alginate–perfluorooctyl cross-linked; (ALG)_net_—alginate cross-linked. The values are presented as mean ± SD (*n* = 6).

**Figure 6:**
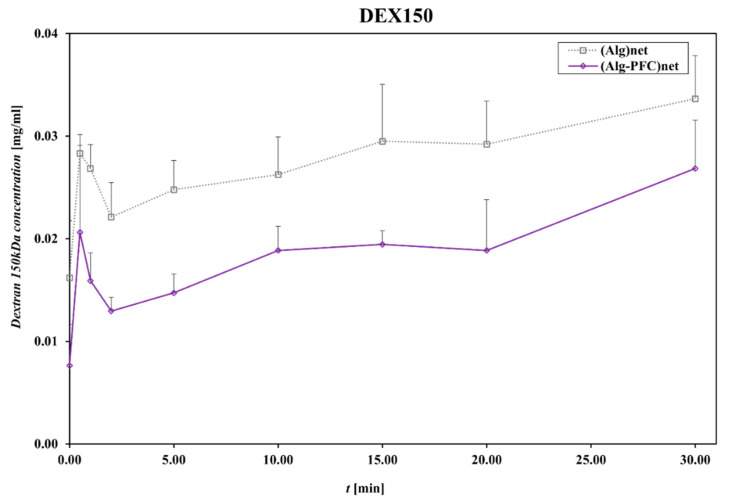
Dextran150 concentration in the function of time during permeation through the membrane. Key to symbols: (ALG-PFC)_net_—alginate–perfluorooctyl cross-linked; (ALG)_net_—alginate cross-linked. The values are presented as mean value ± SD (*n* = 6).

**Figure 7:**
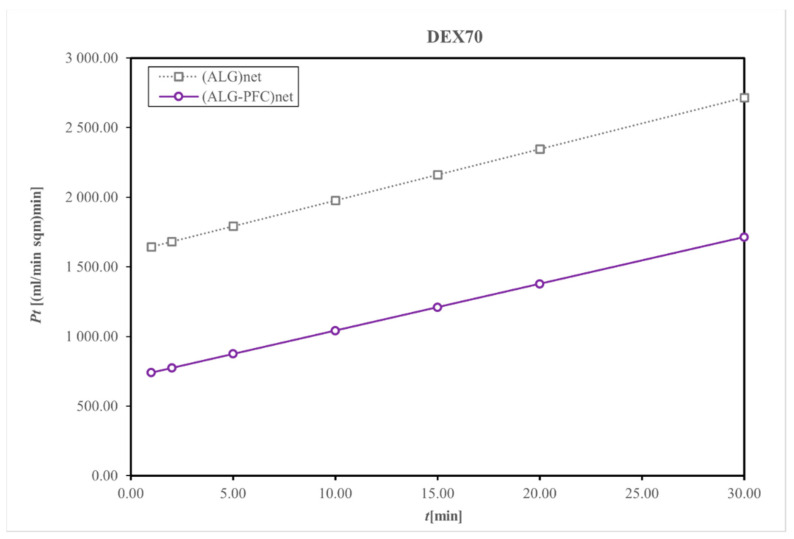
Representative membrane permeability and time product during particular time intervals permeation for Dextran 70. Key to symbols: (ALG-PFC)_net_—alginate–perfluorooctyl cross-linked; (ALG)_net_—alginate cross-linked.

**Figure 8:**
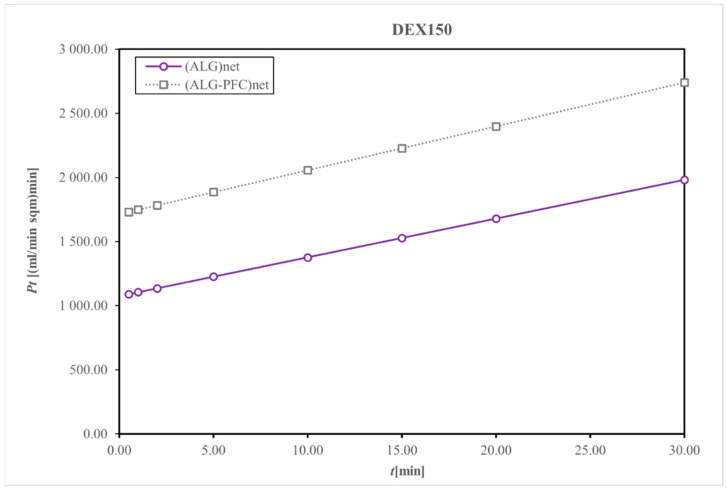
Representative membrane permeability and time product during particular time intervals permeation for Dextran 150. Key to symbols: (ALG-PFC)_net_—alginate–perfluorooctyl cross-linked; (ALG)_net_—alginate cross-linked.

The membranes (ALG-PFC)_net_ and (ALG)_net_ exhibited relative changes in the concentration of model particles during the examination time (Figure 5 and Figure 6). The changes for particular time intervals of permeability and time product confirmed membrane permeating ability for examined solutes of molecular weight 70 and 150 kDa (Figure 7 and Figure 8). Comparing the thermodynamic behavior of sodium alginate and alginate with PFC, it was observed that PFC involvement does not influence transport through the alginate membrane.

Moreover, the membranes do not delimit the transport of the albumin (70 kDa) and immunoglobulin levels (150 kDa).

### 3.4. Fourier Transform Infrared (FT-IR) Spectroscopy 

**Figure 9:**
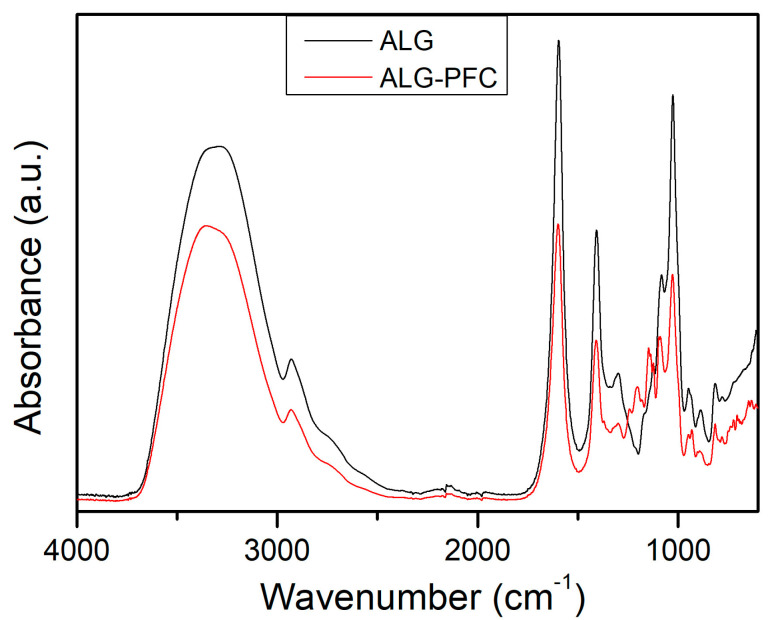
FTIR spectra of sodium alginate (ALG) and sodium alginate with perfluorooctyl (ALG—PFC).

The Fourier transform infrared spectra of sodium alginate and sodium alginate with perfluorooctyl are shown in Figure 9. Spectrum of sodium alginate exhibited absorption bands regarding hydroxyl, ether, and carboxylic functional groups. Stretching vibrations of O–H bonds of alginate (which appeared in the range of 3000–3600 cm^−1^) were observed at 3329 cm^−^^1^. The bands observed at 1600 and 1414 cm^−1^ can be attributed to stretching vibrations of the carboxylate group. The band at 1043 and 1086 cm^−1^ is attributed to the C–O stretching vibration. After oxygenating factor involvement in the alginate, the bands at 1143 and 1200 cm^−1,^ which can be attributed to C-F bonds, appeared. 

### 3.5. Evaluation of Morphology of Oxygenated Membrane and the Cells within the Membrane

**Figure 10:**
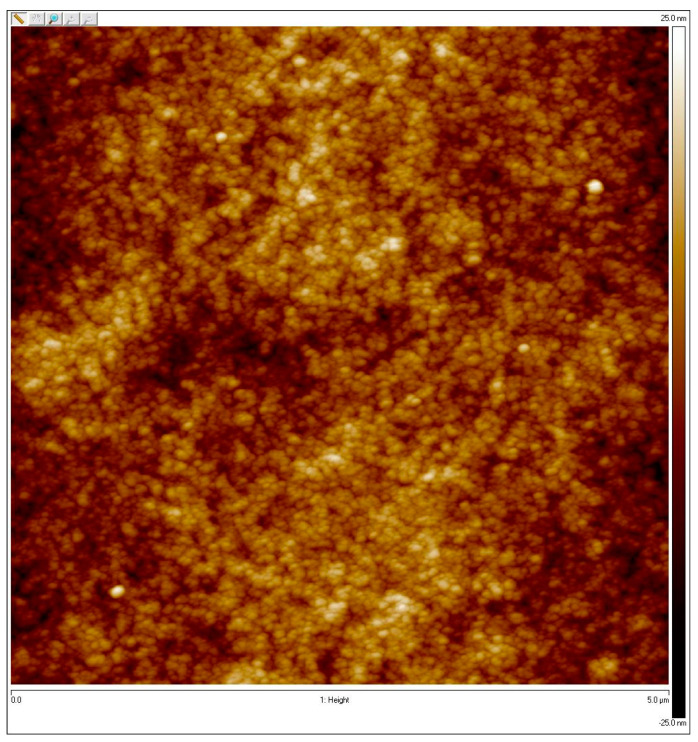
AFM visualization of (ALG—PFC)_net_ layer deposited on the gold mica substrate cover.

In order to characterize the surface topography of the prepared membranes, atomic force microscopy (AFM) was employed. AFM visualization of the applied (ALG-PFC)_net_ layer deposited onto gold-covered mica substrate is presented in Figure 10. The effect of a conglomeration of the polyguluronic acid segments in clusters due to cross-linking can obtain the even structure of the coating layer. As a result, the granular branched structure with an average grain size of 5 nm (based on a particle analysis tool) can be observed. In addition, the root means square roughness parameter for the whole surface was measured to have a 5.5 nm value. 

**Figure 11:**
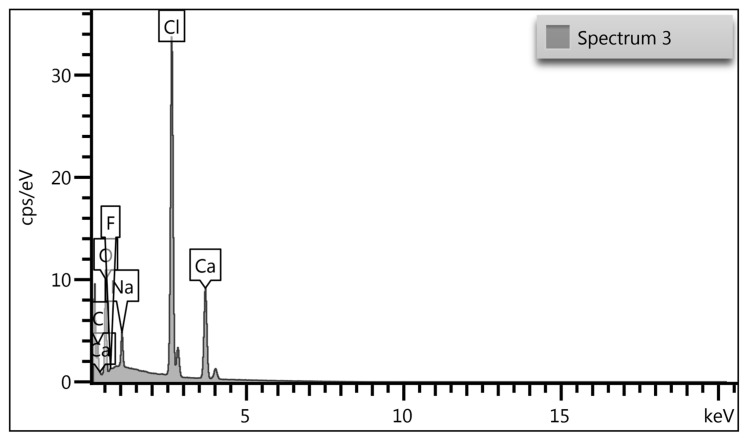
EDX spectrum of the sample (ALG-PFC)_net_.

Chemical composition analysis of the cross*-*linked (ALG-PFC)_net_ samples by X-ray spectrometer (EDX) was performed using an EDX analyzer. (ALG-PFC)_net_ mapping images in the spectra show the characteristic elements for perfluorooctyl and the cross*-*linking factor. There were mean values obtained: O: 11.88 ± 4.50; F: 0.25 ± 0.01; Ca: 26.42 ± 3.55; Cl: 51.61 ± 6.47. A representative image of the spectrum is presented in Figure 11. The obtained results confirmed the presence of oxygenating and cross-linking factors. 

**Figure 12:**
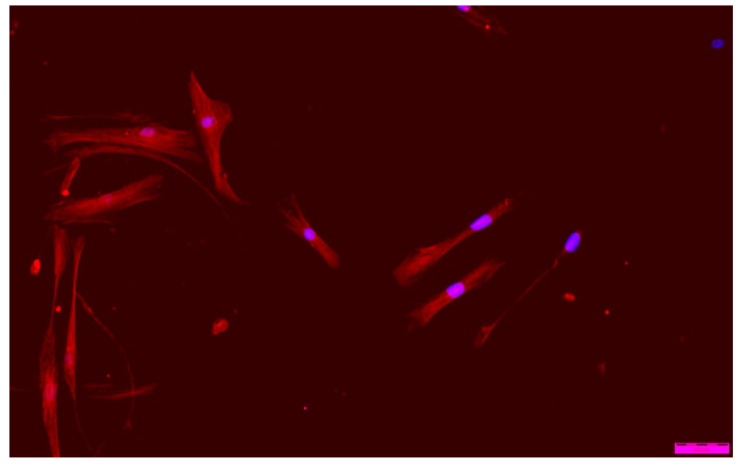
Fluorescence microscopy image of human fibroblast cells cultured in the presence of the alginate–perfluorooctyl (ALG-PFC) coating after a 6-day culture.

**Figure 13:**
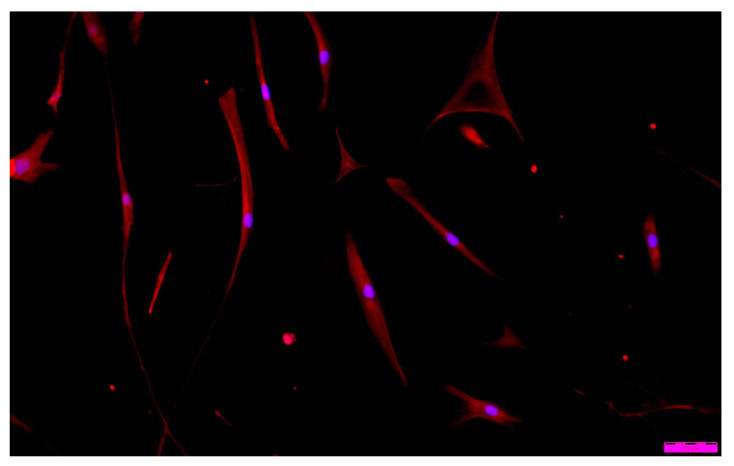
Fluorescence microscopy image of human fibroblast cells cultured in the presence of a cross-linked alginate–perfluorooctyl coating ((ALG-PFC)_net_) after a 6-day culture.

When analyzing ‘cell’ morphology using fluorescence microscopy, it was observed that cells grown in the presence of ALG-PFC and (ALG-PFC)_net_ membranes show the correct morphological picture (Figure 12 and Figure 13). After six days of the culture, we observed numerous cells on the surface of glass coverslips coated with ALG-PFC and (ALG-PFC)_net_. In addition, the spindle-shaped cells of fibroblastoid features were present on both types of surfaces. 

## 4. Conclusions

In summary, PFC involving the alginate layer improved HDF function during the 6 days of the experiment, influencing viability and mitochondrial activity without delimiting the permeability. Since PFC is immiscible in aqueous solutions, including blood, it can constitute a safety factor of the dressing element at the interface with the wound. Moreover, the layer coating material involving the oxygenating and cross-linking factors topography ensures an even contact surface for interface with the wound. Applying the oxygenating and/or cross-linking factors causes fluctuation of mitochondrial activity during the culture on the modified support patches. Concerning these fluctuations, the systems of modified patches can be recommended for application up to six days (five days of contact of cells with modified patches). Sodium and calcium alginates are widely used as dressings. Although they are not the healing components, such covers do not adhere to seeping wounds, and because they are highly hydrophilic, they absorb exudate and retain moisture, promoting healing. Therefore, the layer coating with applied oxygenating configuration within alginate can involve a therapeutic function to the element of the dressing platform.

Generally, the ideal universal wound dressing still has not been provided; thus, new products have been developed and functionalized to obtain the properties that can influence the wound-healing process. Such a product can be the polyelectrolyte alginate layer coating with an incorporated oxygenating factor, which can be recommended as an element of a dressing system.

## Data Availability

Not applicable.
